# Why Anthropologists Should Study Dogs

**DOI:** 10.1002/evan.70050

**Published:** 2026-10-14

**Authors:** Kyle C. M. Smith

**Affiliations:** ^1^ Department of Anthropology, College of Liberal Arts Pennsylvania State University University Park Pennsylvania USA; ^2^ Department of Clinical Sciences, College of Veterinary Medicine North Carolina State University Raleigh North Carolina USA

**Keywords:** bioarchaeology, convergent evolution, cultural variation, domestic dog, human evolution, research ethics, social cognition, social determinants of health

## Abstract

While primates have traditionally been a focus of evolutionary anthropologists, the domestic dog represents a valuable complementary model due to its long shared evolutionary history with humans. The advantages of studying dogs are particularly evident in four key areas: (1) analyzing evolutionary adaptations, (2) understanding humanity's past, (3) examining humanity's cultural diversity, and (4) assessing factors that influence human health. Dogs have convergently evolved social cognition and local adaptations (including altitude adaptations and the ability to digest starches and lactose) that parallel those of humans, making them valuable models for testing evolutionary hypotheses. The global spread of dogs also illuminates human population histories, and isotopic analyses of dog remains can be proxies for human dietary data when sampling human remains is infeasible or inappropriate. Dogs' near‐universal presence across human societies and exposure to the same real‐world environments further make them uniquely powerful models for studying cultural variation and health.

Evolutionary anthropology brings an evolutionary framework to the study of humanity in all its complexity. Not only does this include studying the evolved biological adaptations of humans, but it also informs sister disciplines like archeology and cultural anthropology by emphasizing population histories and the biological and cultural adaptations to varying conditions of life over time and around the world. While evolutionary anthropology has traditionally emphasized comparing humans to our closest relatives, the primate order, I argue that the field could also benefit from studying the animal that has had the longest evolutionary history alongside humans: the domestic dog (*Canis familiaris*). Specifically, I identify four goals of anthropology where I argue that studying dogs may be particularly fruitful: (1) analyzing evolutionary adaptations, (2) understanding humanity's past, (3) examining humanity's cultural diversity, and (4) assessing factors that influence human health. I conclude by discussing some limitations and the ethical and practical considerations of this proposal.

## 1 Analyzing Evolutionary Adaptations

A key focus of evolutionary anthropology is the study of how and why particular human adaptations emerged as well as the fitness trade‐offs and biological mechanisms underlying these traits. As dogs live in most of the same environments as humans, they have convergently evolved similar adaptations, such as to diets containing dairy and starch and to high altitudes. Dogs are also increasingly used as a model for human cognitive evolution [1, 2].

Dogs have converged with humans evolutionarily in their adaptations to particular diets, such as starch and lactose. For instance, humans have evolved an increased copy number of *AMY1*, the gene for salivary amylase, relative to great apes, with agricultural societies exhibiting higher copy numbers on average due to greater starch consumption compared to forager and pastoralist societies [3]. Similarly, dogs have increased copy number of *AMY2B*, the gene for amylase in the pancreas, compared to wolves [4]. Dogs from regions with limited starch consumption, such as the Arctic, have fewer copies of this gene than those from areas where starch is more commonly consumed [5]. Humans in various societies that consume dairy have evolved the continued production of lactase, the enzyme that breaks down lactose, into adulthood [6]. Dogs in Europe and the Middle East have also undergone positive selection for an allele of lactase that likely increases expression of this gene [7].

These patterns in dogs can be useful for testing hypotheses for human dietary evolution. While the associations of amylase copy number with agriculture and of lactase persistence with dairying are well established, it is still debated which exact selective pressure favored these genes [8, 9]. Amylase has a limited role in starch digestion but is also involved in the stress response and expressed in mammary glands, seminal fluids, and the uterus, so it has been suggested that these latter roles could be implicated in the selective pressure [9]. Some populations, such as central Asians, have high levels of dairy consumption with low levels of lactase persistence [8], and dairy consumption existed for thousands of years in Europe before lactase persistence was selected for [10]. It has been suggested that particular forms of dairy consumption, such as a reliance on fermentation to partially break down lactose before consumption, could reduce the selective advantage of lactase persistence in certain societies [8], or perhaps that lactase persistence only exerts a strong effect on fitness under conditions of high nutritional or pathogen stress [10]. Examining the patterns of when and where dogs evolve lactase persistence could be particularly helpful in disentangling the selective pressures involved [11].

Evidence from dogs also suggests that the microbiome could play a key role in allowing dietary expansion prior to the initiation of selection on digestion. For instance, ancient DNA from Bronze Age Italian dog coprolites has revealed that these dogs had not yet evolved extra copies of amylase, but their gut microbiomes had microbes that produced alpha‐amylase at levels well beyond those seen in modern dogs [12]. This suggests a pathway by which dietary evolution can occur in dogs and other species: changes to the microbiome can allow an animal to digest new foods enough for them to be a component of the diet, and then over time, this opens up the possibility for natural selection to operate on the host animal's own genetic adaptations to the diet. It is possible this pathway could also occur in humans. Individual humans who consume dairy but are not lactase persistent have higher levels of *Bifidobacterium*, which can break down lactose [13], so perhaps (like dogs’ adaptations to starches) humans adapted to consuming lactose initially via the microbiome and then later by their own genetic evolution. Further exploration of this process in dogs may prove illustrative for how such adaptations can occur in humans.

Another example of the convergent evolution of dogs and humans takes the form of adaptations to high altitudes. In high‐altitude regions such as Tibet, both dogs and humans have adapted to chronic hypoxia via modifications to the *EPAS1* gene, which codes for hypoxia‐inducible factor‐2*α* [14]. This gene was selected for after introgression from populations that had been present in the region for longer: Denisovans in the case of humans and gray wolves (*Canis lupus*) in the case of dogs [15, 16]. Human populations have evolved adaptations to high altitudes in Tibet, Ethiopia, and the Andes, and various specific adaptations are present, including increased alveolar ventilation, enhanced pulmonary oxygen diffusion capacity, and higher hemoglobin concentrations [17]. By studying the altitude adaptations of dogs in different parts of the world and comparing these to human adaptations, we can better understand the fitness trade‐offs and mechanisms underlying the various evolved adaptations to chronic hypoxia in humans.

The example of altitude adaptations in Tibetan humans and dogs raises another interesting similarity in the two species’ evolution: the importance of adaptive introgression from other populations or species. As anatomically modern humans spread from Africa to Eurasia, they admixed with Neanderthals and Denisovans, and the introgressed variants had functional consequences for traits such as the immune system and circadian rhythms [18]. Likewise, adaptive introgression has been important in dog evolution. Most modern dogs show evidence of admixture from wolves, with evidence that this has had an effect on olfactory receptors in particular [19]. Recent evidence suggests that two genes involved in dogs’ starch digestion, *AMY2B* and *MGAM*, may have been introduced to dogs from African wolves (*Canis lupaster*) [20] or possibly from an unknown divergent canid in the case of *AMY2B* [21]. Adaptive introgression can be particularly likely when a population expands its range and has to adapt to novel conditions that other local populations have already adapted to [22]. As multiple copies of the beneficial allele can be introduced at once, the probability of fixation is higher than for novel mutations [23]. Both humans and dogs dispersed globally, including into regions where other species from the same genus had already resided for a long time, so it is particularly likely that they were able to pick up beneficial alleles through admixture to help them adapt to the novel environments they encountered. Dogs have been known to interbreed with gray wolves [19], African wolves [24], Ethiopian wolves (*Canis simensis*) [25], coyotes (*Canis latrans*) [26], golden jackals (*Canis aureus*) [27], and even with the pampas fox (*Lycalopex gymnocercus*) [28]. Future research should continue to explore the degree to which there has been introgression from these canids and any parallels with hominid evolution. In particular, as Ethiopian wolves are genetically adapted to high altitudes [29], these adaptations may have been particularly likely to have introgressed into dogs.

Dogs began to attract some attention in anthropology due to the finding that their comprehension of pointing gestures exceeds that of both their closest relatives (gray wolves) and humans’ closest relatives (chimpanzees, *Pan troglodytes*) [30]. However, debate around this topic grew due to a rising number of studies finding that various species, including non‐primates and non‐domesticates, can follow human pointing gestures above chance [31]. Several studies have subsequently tried to compare dogs and wolves directly on their comprehension of pointing [32, 33], but even re‐analyses of these same datasets have caused disagreements over whether to interpret the findings as indicating that dogs outperform wolves, with debates over the role of socialization and experience, which experimental designs are appropriate, and the role of temperament versus cognition in determining task performance [34, 35, 36]. The behavioral development of dogs and wolves is complicated by the interactions between their genetics and environments. For instance, wolves have their critical period of socialization at younger ages than dogs, such that dogs experience their critical period with all their senses developed, but wolves only have olfaction for the first half of their critical period. This could result in wolves’ initial familiarization with the smell of conspecifics limiting their subsequent interspecies interactions in a way that does not happen in dogs, setting up species differences in later interactions with and attention to humans [37]. One thing missing from the debate about dogs following human pointing is attention to the cross‐cultural variation in how humans point. While pointing with the index finger has been claimed by some to be a human universal, it is not the most commonly used form of pointing in all societies, with some cultures preferring to point with the lips, eyes, or nose [38, 39]. Future research should examine whether dog populations living with humans who primarily point non‐manually are more attendant to the locally‐preferred gestures.

The propensity of dogs to communicate with humans extends beyond gestural communication. When presented with a task they cannot solve on their own, dog puppies make more eye contact with humans than wolf pups do [33, 40]. Experimental evidence indicates that dogs can distinguish between positive and negative emotional valences in human facial expressions and vocal tones [41]. A comparison of individual differences in the cognition of dogs, chimpanzees, and human infants suggests that the underlying structure of cognitive differences is more similar between human infants and dogs than humans and chimpanzees, with social tasks clustering separately from physical reasoning tasks in humans and dogs but not in chimpanzees [2]. Dogs may also be an informative model for understanding the evolution of language, as dogs around the world have been trained to respond to specific commands when hunting [42], herding [43], and hauling sleds [44], and dogs have in some cases been able to learn over one thousand words [45]. The growing popularity of button‐based soundboards among pet dogs is also providing novel opportunities to look at dogs’ communicative abilities [46].

Some researchers have hypothesized that both humans and dogs have undergone convergent evolution due to selection against aggression, a concept referred to as the human self‐domestication hypothesis [1, 47]. As early as Darwin, it has been noted that particular traits tend to recur in domesticated animals compared to their wild counterparts, such as reduced fear and aggression, smaller teeth, shorter snouts, and increased frequency of floppy ears and tails and of depigmented patches [48, 49]. This “domestication syndrome” has drawn attention in part due to a long‐running experiment in which red foxes (*Vulpes vulpes*) were bred for reduced aggression and subsequently developed various other traits of the syndrome [50]. The human self‐domestication hypothesis proposes that humans have undergone selection against aggression, which as a result, caused elements of the domestication syndrome, such as reduced prognathism and smaller teeth [1, 51]. While this hypothesis has been criticized as lacking sufficient evidence and clarity, it generates testable predictions and can continue to be evaluated [52].

Future research can utilize the growing number of available dog genomes to test for signatures of selection. The Dog10K consortium has published around 2,000 dog genomes, including over 300 breeds and village dogs from two dozen countries, and they plan to increase this sample to 10,000 [53]. Additionally, thousands of other dog genomes are publicly available through the Dog Aging Project and Darwin's Ark projects, each with a variety of associated metadata [54, 55]. The growing number of ancient DNA sequences from archeological dog specimens, including those older than 15,000 years [56], will also make it possible to observe genetic changes over time more directly.

## 2 Understanding Humanity's Past

The collaborations of evolutionary anthropologists and archeologists can reveal human population histories as populations migrate and mix, and reconstructing past lifestyles is important for understanding the evolutionary pressures shaping humans. Dogs provide several unique opportunities due to their long history alongside humans across diverse societies. The study of dogs allows more nuanced insights into human migrations and interactions between different populations. Additionally, in some cases, dogs' remains may serve as a proxy for human remains in destructive analyses, offering an ethical alternative when such studies would otherwise be inappropriate for human samples.

Dogs were domesticated in Late Pleistocene Eurasia, though the precise location, timing, and number of domestication events is subject to debate [57, 58, 59, 60, 61]. This makes dogs the first domesticated animal and the only one to predate agriculture [62]. Once domesticated, dogs spread to almost all human societies, adapting to diverse climates and cultural contexts (Figure 1). After their initial domestication, dogs quickly spread across Eurasia, and they eventually reached human communities in Africa and Australia [64, 65]. They also accompanied human migrations into the Americas [67], Polynesia [66], and the Arctic [63].

**Figure 1:**
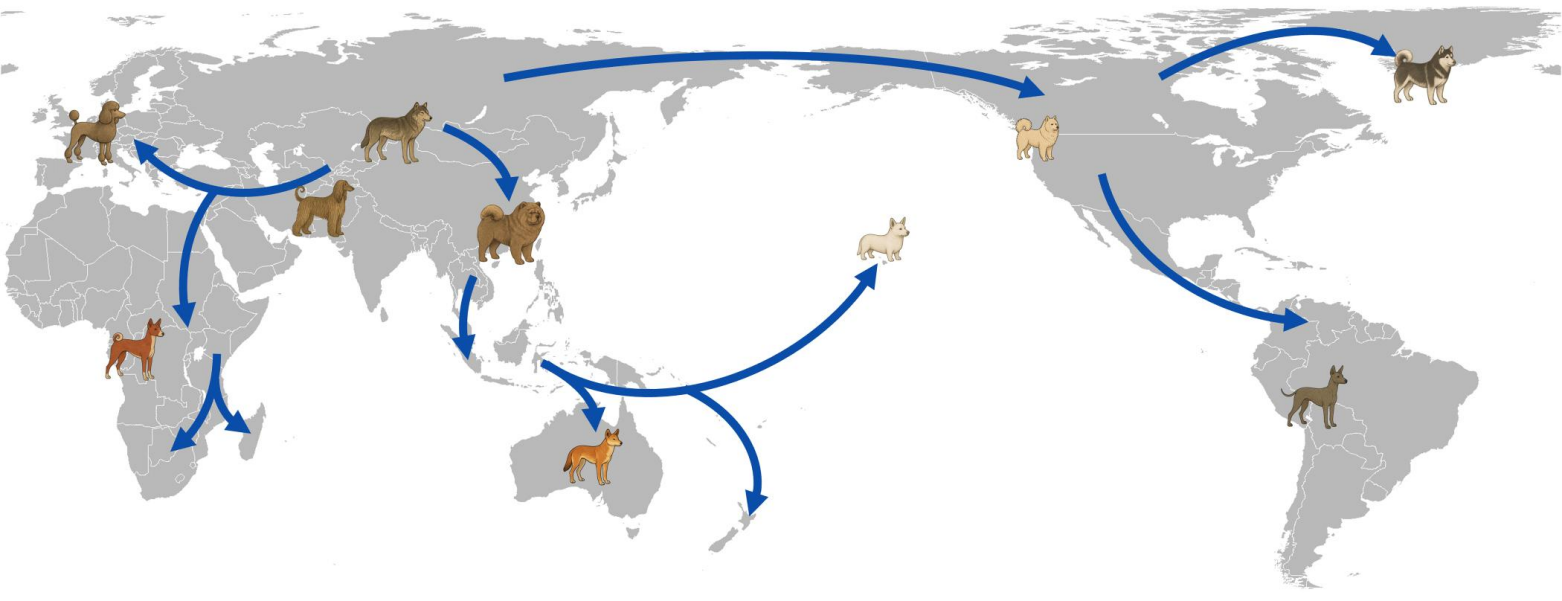
A map of the approximate route of dog dispersal around the world, with distinctive local breeds depicted. Routes are based on information from the published literature [63, 64, 65, 66, 67].

This global spread of dogs means that dogs can be used to trace human migrations and provide evidence for past contacts between societies. For instance, it is debated whether there was gene flow from Asia into Australia in the mid‐Holocene [68]. While dogs cannot definitively resolve that debate, it is relevant that the archeological and genetic evidence clearly demonstrates that Australian dingoes and New Guinea singing dogs arrived across the Wallace line from Asia in the mid‐Holocene, far later than the original human settlement of these regions, therefore proving at least some degree of contact between these continents [65]. Future ancient DNA sampling of dingoes and southeast Asian dogs will help clarify when and from where dogs were introduced into Australia and New Guinea. Dogs can also be used to help trace human migrations. For example, ancient DNA from dogs provides similar estimates to genetic data from humans about the timing and pathway of humans' settling of the Americas [67] and of Neolithic farmers’ migration into Europe [69]. A comparison of dog and human genomic data, however, can be illuminating for both their similarities and their differences. While both human and dog genomes indicate an influx of Southwestern Asian ancestry into Europe in the Neolithic, subsequent populations of humans derive lower proportions of their ancestry from the previous forager populations of Europe than dogs do from that previous population's dogs [70]. Besides genetic data, cultural data related to dogs can also be used in this manner. A phylogenetic analysis of the similarities between dog‐related mythology of various cultures found a tree rooted in Central Asia, aligning with the hypothesized origin of domesticated dogs and suggesting such myths may trace back to this time [71].

Bioarchaeological examination of dog remains can provide insights into past human societies and the nature of dog‐human interactions. All around the world, there are examples of dog remains that have been buried in a manner showing similar care and attention as that given to human remains, which can suggest the emotional relationships between humans and dogs [72, 73]. However, just because dog remains are found in archeological deposits does not necessarily imply a deliberate burial intended to show respect for the dog, as a burial could also be a convenient way to dispose of a corpse, or dogs could be sacrifices interred as grave goods [74, 75]. Skeletal pathologies can also be informative as to the relationship between dogs and humans. One of the earliest unambiguous dog skeletons, the Bonn‐Oberkassel dog (found in Germany and dated to around 14,000 years ago), shows evidence of severe pathologies that healed, which suggests that by this time humans were already providing care for dogs [76]. Some skeletal pathologies on Arctic and North American dogs are potentially indicative of the heavy load‐bearing involved in hauling a sled or travois [73]. Dogs are consumed as food in some cultures, and post‐mortem indicators of butchery on their bones can be evidence of this [75].

Archeologically, isotopic analyses of dog remains can provide information about human diets. Stable isotope analysis can reveal dietary differences between individuals and populations, but there can be ethical concerns in applying this to human remains, particularly when they are rare or when descendant communities oppose such practices. Destructive sampling of dog remains may be less controversial alternative, provided that dog diets closely reflect those of the humans they lived alongside. Some have advocated for the Canine Surrogacy Approach, or the use of dogs as dietary proxies for humans in stable isotope analyses, on the grounds that the results between sites have generally been similar, with some exceptions [77]. However, caution should be taken when applying this approach. The scale of analysis matters, as in living horticulturalists, dog isotopes have been found to be reasonable proxies for human diets at the site level, though less so at the household level [78]. The possible misidentification of wild canids as dogs can lead to inaccurate inferences [77]. Some dietary differences between dogs and humans, such as feces consumption by dogs, the consumption of dog meat by humans [77], or taboos on human consumption of some foods given to dogs [44], may provide cultural insights through the isotopic differences between humans and dogs. Perhaps the situation in which dog remains can be the most strongly informative about human diets is in environments where, for instance, the predominant C4 plants available are domesticates such as maize, so signatures of C4 plant consumption in dog remains can indicate a society that produced such plants [79].

## 3 Examining Humanity's Cultural Diversity

Not only can humans adapt to different environments through their genetics, but culture is also an important mechanism of adaptation. Studies of human cultural variation can take advantage of the near‐universal presence and versatile social roles of the dog in order to examine the patterns of cultural diversity. Dogs frequently have roles as hunters, herders, haulers, and companions, making them well suited for both comparative and intensive cultural study.

Hunting is one of the oldest and most widespread functions that dogs have served in human societies [80]. Understanding the role of dogs as hunting companions facilitates the testing of models of human foraging decisions. For instance, optimal foraging theory has been used as an evolutionary framework for modeling human foraging decisions, predicting that foragers will target food items that maximize energy gained per unit of time spent searching, handling, and processing [81]. Research has shown that dogs can either increase or fail to increase hunting efficiency, depending on factors such as prey type, habitat, and other environmental factors [82, 83, 84]. Dogs improve hunting efficiency by reducing search and handling time through their ability to detect, track, pursue, flush out, trap, capture, and kill prey [80, 83]. Through the use of the optimal foraging model and an empirical understanding of how dogs reduce search and handling costs, anthropologists can make sense of why people focus on the prey they do in a particular cultural context. For instance, in the early Holocene, dog burials by the Jōmon culture have been found to spatially and temporally correlate with evidence of the hunting of forest ungulates, which has been interpreted as indicating that dogs played a key role in assisting humans in hunting this prey [85].

The ethnographic record provides a few interesting case studies in which dogs were introduced to societies in historic times and may have had a pivotal role in precipitating cultural changes. In Tasmania and the Andaman Islands, dogs were absent until they were introduced by European colonists in the 19th and 20th centuries [86, 87]. In both locations, dogs came to outnumber humans within a few decades and were rapidly integrated into these societies [86, 87]. Ethnographers noted that the Andamanese and Tasmanians became very fond of dogs, gave them personal names, and even breastfed them [86, 87, 88]. Dogs became important hunting companions, which in the Andaman Islands has been argued to have played a role in people reducing their reliance on shellfish in favor of hunting wild boars, which were much easier to hunt with dogs [86].

Besides hunting, dogs have made many other tangible contributions to human societies. Dogs’ keen sense of smell and hearing makes them valuable as watchdogs, capable of alerting and defending humans from wild animals and from potentially hostile strangers [89]. In societies that rely on livestock, dogs may function as guardians protecting herds from predators or as herders directing livestock [90]. As dogs improve the ease of managing large herds, their presence may have been a strong advantage to herders. It has been estimated that a nomadic shepherd can manage 300 to 400 sheep or goats without a dog but up to 500 with a dog [91], but future work should better quantify the difference dogs make to pastoralists under various conditions. Dogs have in some regions been useful for transportation, pulling sleds through the Arctic [92] and carrying packs and travois in the American Great Plains [93]. Dog consumptions as food has been traditionally practiced in various societies worldwide, but strong cultural taboos against eating dogs are also globally widespread [94]. In at least one society, dogs were raised for fabric production: the Coast Salish people of the North American Pacific Northwest traditionally bred a unique woolly dog whose long white hair was woven into blankets and other textiles [95].

In addition to fulfilling concrete functions, dogs also serve as social companions to humans. Around 85‐94% of American dog owners consider their dogs to be a member of the family [96]. However, viewing dogs as social companions and akin to humans is neither idiosyncratic to modern Western societies nor universal across cultures or throughout history. Different cultures categorize the world in diverse ways, and the terms “human” and “person” are not always viewed as synonymous: some cultures reserve personhood exclusively for humans, while others extend it to a broad range of entities, with some cultures grouping dogs as more similar to humans than to other animals [97, 98]. Across various societies, dogs have been given individual names, considered part of the kin group, provided with burial practices otherwise reserved for humans, and even allowed to sleep alongside humans in their beds [98]. Conceptualization of kinship in human societies is always related to genetic relatedness but never exactly synonymous with it, which has led to the hypothesis that kinship best understood as a representation of fitness interdependence [99]. Cross‐cultural comparisons of dogs provide an opportunity to test this hypothesis, as dogs are sometimes considered as kin and can have interdependent fitness with humans without close genetic relatedness.

There is great potential for cultural anthropologists to study dogs in both broad and intensive ways. For instance, at least two authors created databases of cross‐cultural data on dogs from the Human Relations Area Files, which will be valuable resources for studying variation in dogs’ societal roles [98, 100]. More intensive research can examine dogs in a particular culture, such as recent research on Salish woolly dogs that merged ancient DNA, isotopes, interviews of descendant communities, excavations, and historical records to contextualize the role of these unique dogs [95, 101].

## 4 Analyzing Factors That Influence Human Health

Human health is a central anthropological focus because it is critical to an individual's fitness and therefore of interest to evolutionary anthropologists, and because improving global health is a key goal of applied anthropologists. Dogs are an underappreciated model for studying human health with unique advantages due to the fact that they live alongside humans across a wide variety of shared environmental conditions.

Research on human health often benefits from studying non‐human animals as models. Longitudinal studies of humans can take decades to reveal chronic health outcomes. The complexities of human societies introduce many confounding variables, and some experimental interventions are infeasible in humans. Mice (*Mus musculus*) are a popular model organism due to their short lifespans and the possibility of tightly controlling and manipulating variables in the laboratory, but there are limitations to how well laboratory findings can be translated to the real world. Field observations of nonhuman primates can provide insights into human health in a naturalistic setting, but they do not experience the full spectrum of environments and lifestyles that humans do, and they also have long lifespans. Uniquely, dogs inhabit the same environments as humans across a comparable diversity of conditions, making them particularly suitable models for studying human health. Like humans, dogs experience variations in lifestyle factors that are relevant to health, including diet, exercise, social interactions, chemical exposures, pathogen and microbe exposures, climatic conditions, and access to healthcare. A comparison of causes of death in U.S. humans and the diagnoses at the time of death in U.S. pet dogs found that the pathophysiological processes, organ systems, and cancer types occurred at similar rates in both species (with the notable exception of vascular causes of death, which are common in humans but rare in dogs) [102]. Furthermore, both species’ age trajectories of mortality (scaled for lifespan) were similar, as were the numbers of comorbidities associated with chronic health conditions [102]. These parallels suggest that dogs are a promising model for how environmental factors affect health and aging [102, 103].

Dogs also provide a good model for testing hypotheses regarding how social factors affect health. The social determinants of health framework, which examines how social factors like social status, social connectedness, and early life adversity affect health and longevity, has become an important area of study in anthropology [104]. This framework has now been applied to pet dogs, revealing correlations between dogs’ health and the family's socio‐economic status and the number of other dogs in the household [105]. While this study was cross‐sectional and could not eliminate other possible confounding factors (e.g., a more invested owner providing better care for more dogs), these findings align with the larger body of research that social relationships can affect health. Future research should continue to explore how the social relationships of dogs affect their health. Chronic stress and its effects on health also present a meaningful area of investigation in dogs. In humans, lower socioeconomic status has been linked to higher rates of various health conditions, with stress related to low status being a proposed contributing factor [104, 106]. In pet dogs, fear of strangers has been associated with significantly shorter lifespans [107], further supporting the importance of stress in health outcomes across species. Future research on dogs should examine this in more detail. For instance, social isolation or low social status in humans and rhesus macaques (*Macaca mulatta*) is associated with changes to gene expression in which antiviral functions are downregulated and antibacterial functions are upregulated [108, 109]. Determining whether this occurs in dogs, for instance when a dog is adopted from a shelter into a stable home, may help determine why such changes occur, such as whether they are widespread in mammals or whether this is the result of changes to the degree of interactions with conspecifics and therefore the risk of viral infections.

Dogs may also be an important model for the role of the microbiome in health. At least on the skin, dogs and humans from the same household have been found to share microbes [110]. Microbes in the gastrointestinal tract and oral cavity may be particularly important given their associations with various health conditions, possibly via microbes and their metabolites translocating throughout the body and contributing to disease risk [111, 112]. The microbiomes of dogs appear to parallel those of humans in some important ways. A signature of domestication in the gut microbiome has been identified in various pairs of related domesticated and wild species, including dogs relative to wolves. Dietary swaps and comparisons to human populations indicate that these adaptations may be linked with an industrialized diet [113]. Like humans, dogs in industrialized societies have less diverse gut microbiomes than those in non‐industrialized societies [114], and like humans, dogs living in industrialized societies have detectable taxonomic differences from those living in pastoralist or forager societies [115].

Future research using dogs as a model for human health will have several large‐scale longitudinal databases to draw from. The Golden Retriever Lifetime Study tracked 3,000 Golden Retrievers across their lifespans, collecting a wide variety of detailed data about them [116]. The ongoing Dog Aging Project is currently collecting genetic, environmental, and health data from thousands of purebred and mixed‐breed dogs [54]. These datasets provide opportunities to test the effects of various environmental factors on long‐term health outcomes. The nature of pet dogs also creates opportunities for randomized clinical trials. Interventions that would take decades to evaluate in humans, or be ethically or practically infeasible, may in some cases be testable in dogs on a shorter timescale. However, researchers studying dogs should follow additional ethical considerations beyond those usually involved in animal research (Box 1). As in some cases it can be hard to understand the role of genetics versus environmental factors when health conditions recur within a family, the presence of non‐genetically related dogs in the household provides a potentially illuminating basis of comparison.

Box 1. **Ethical considerations in dog research**

When studying dogs, investigators should of course follow all relevant ethics oversight requirements for working with animals. However, dog researchers would benefit from considering the ethical guidelines for human infant research. While children and infants have limited autonomy and ability to comprehend and consent to research, they should nevertheless be given the opportunity to choose whether or not to participate in research, to the extent that their cognitive abilities allow [117]. In a similar vein, much non‐invasive research involving dogs can be designed so that subjects can opt not to participate. Just as research with underage children requires the informed consent of a parent or legal guardian, a pet dog's legal owner should provide informed consent. When studying dogs in different cultural contexts, it is vital to be sensitive to the specific role that dogs play within those societies. For studies of free‐ranging dogs, the appropriate model is non‐invasive animal research, involving obtaining local and national permissions and minimizing any disturbance to the animals.

## 5 Limitations and Suggestions

As anthropologists are well aware, most psychological and medical research on humans is done in industrial societies, primarily in North America and Europe. However, these societies are not representative of the diversity of human lifestyles and differ in substantial ways from how humans lived for most of our species’ existence [118]. Likewise, most dog research studies companion dogs in North America and Europe, but these differ from the majority of dogs in several important ways: they are typically sterilized, receive veterinary care, eat commercial dog food, undergo obedience training, have a recent history of intensive breeding, and live relatively isolated in houses [119]. Just as anthropologists study humans in diverse settings to more fully understand the human experience, researchers should expand their focus to include dogs living in diverse contexts, including dogs in societies with more traditional lifestyles, free‐ranging and feral dogs with limited human interaction, and dogs performing jobs such as hunting and herding. By studying dogs in a broader range of environments, we can understand more comprehensively how dogs interact with humans, adapt to different ecological and social conditions, and contribute to human societies. In addition to studying dogs around the world, fruitful comparisons can be made between dogs and their relative the gray wolf, with dingoes (which descend from domesticated dogs but now breed in the wild), with canids like coyotes and red foxes that have commensal urban populations, and with experimentally domesticated foxes that have converged with dogs [50].

In the US, unlike in much of the world, most pet dogs are spayed and neutered [119]. Studying sterilized dogs limits our ability to use fertility as a measure of evolutionary fitness, so other proxies such as health and longevity are more applicable. Sterilizing dogs also limits their utility for researching reproductive health, sex differences, and the influence of sex hormones. However, a sample of both intact and sterilized dogs facilitates testing the effects of sex hormones in a way difficult to do in humans. Western companion dogs also differ from most dogs globally, and from most humans, because their proximate cause of death is frequently euthanasia [120]. This limits directly comparing their health conditions and lifespan with humans. However, euthanasia typically occurs due to underlying health conditions, which are comparable to human causes of death, and the lifespan of euthanized dogs may be a proxy for humans’ duration of healthy living [103].

## 6 Conclusions

The study of dogs offers opportunities for a variety of research topics within evolutionary anthropology. Dogs provide an excellent opportunity to test hypotheses about why particular local adaptations were favored by selection. If similar adaptations exist in dogs, then comparisons about when and where these adaptations were favored, or associations of these traits with fitness, can be compared in dogs and humans. For instance, hypotheses on why lactase persistence was favored in some dairy‐consuming cultures but not others should be able to explain the pattern observed in dogs. There is also growing evidence that dogs have developed unique communication and social bonding abilities with humans, which has implications for understanding the evolution of cooperation and communication. As these are key components of human evolutionary history, researchers focusing on human social cognition should consider turning to dogs as a model organism. Researchers tracking human migrations around the world and trying to understand past contacts between societies can study the migrations of dogs around the world using ancient and modern DNA. Archeologists interested in past diets may want to perform isotopic analysis on dog remains if doing so on human remains is difficult or impossible. Hypotheses pertaining to the evolution of cultural traits, such as hunting patterns and conceptualizations of kinship, can be tested with datasets on dogs’ roles in societies around the world. Furthermore, dogs can also serve as important models for understanding human health. Companion dogs live in human homes around the world and are exposed to many of the same environmental factors as their human counterparts, such as diet, exercise, pathogens, and chemicals. This offers unique opportunities to study health outcomes in a real‐world context, which is often difficult to achieve using laboratory organisms, and large datasets like the Dog Aging Project and Golden Retriever Lifetime Study can be used to answer various questions about dog health. Ethically, anthropologists working with dogs should follow guidelines similar to those used in research involving human infants and children. This would ensure that the treatment of dogs in research is humane, with an emphasis on minimizing harm and respecting their well‐being. Additionally, researchers should aim to study dogs from a diverse range of environments and cultural contexts to fully appreciate the diversity of both canine and human experiences. This approach will help uncover the broad range of factors that influence health, behavior, and evolution in both species.

## Funding

The author has nothing to report.

## Conflicts of Interest

The author declares no conflicts of interest.

## Data Availability

The authors have nothing to report.
